# 肺癌低氧微环境与化疗耐药相关性研究进展

**DOI:** 10.3779/j.issn.1009-3419.2014.03.14

**Published:** 2014-03-20

**Authors:** 洋 郭, 密璐 吴, 君慧 赵, 瑜英 李

**Affiliations:** 1 810001 西宁，青海大学 Qinghai University, Xining 810001, China; 2 810001 西宁，青海大学附属医院肿瘤科 Department of Oncology, Affilitated Hospital of Qinghai University, Xining 810001, China

**Keywords:** 低氧, 肺肿瘤, 化疗, 耐药, Hypoxia, Lung neoplasms, Chemotherapy, Drug-resistance

## Abstract

肺癌是我国近年来发病率最高的恶性肿瘤之一，肿瘤低氧首先在肺癌中被发现，低氧与肿瘤细胞的耐药、凋亡、迁徙、血管生成等有密切联系。化疗耐药是肺癌治疗失败、病情进展的重要原因之一，近年来相关研究较多，现对肺癌低氧微环境以及其诱导化疗耐药作用机制的研究进展进行综述。

## Abstract (en)

Lung cancer is one of the malignant tumors with highest incidence recently in our country. Tumor hypoxia phenomenon was first discovered in lung cancer. It plays important roles in tumor cell drug-resistance, apoptosis, invasion and metastasis, angiogenesis. Chemotherapy-resistant is one of the core reasons of treatment failure and disease progress, and many works on the study of chemotherapy-resistant have been done. This article reviewed the research progress of those mechanisms by which lung cancer hypoxia microenvironment could induce chemotherapy-resistance.

肺癌是我国发病率最高的肿瘤之一，多数患者发现较晚，确诊时已经丧失了手术机会，因此化疗是肺癌治疗的重要手段，但是很多肺癌患者尤其是非小细胞肺癌患者的化疗疗效较差且易耐药。研究证明，实体瘤细胞在低氧环境中对化疗药物的敏感性是下降的，低氧可能是导致化疗耐药的重要因素。

## 1 低氧的特点

肿瘤低氧的概念是在肺癌肿瘤组织研究中首次提出的^[1]^。低氧是大多数实体肿瘤共有的特征，当肿瘤超过一定大小时，肿瘤内部即处于缺氧状态。低氧通过作用于低氧诱导因子（hypoxia inducible factor, HIF）信号通路对肿瘤产生重要影响。HIF是由α和β两个亚基组成的异源二聚体，α亚基是活性亚基，其活性与氧分压的高低直接相关。细胞内氧分压正常时，HIF-α蛋白会被脯氨羟基化酶（prolyl hydroxylase domain, PHD）羟基化，而后被希林佩尔林道基因产物（product of von Hippel-Lindau gene, pVHL）识别并结合，进而被泛素化后降解^[2, 3]^；而在低氧条件下，PHD活性受到抑制，HIF-α被羟基化、识别、降解的过程受到抑制，HIF-α在细胞内大量累积，进一步结合HIF-β，作为转录因子进入细胞核与低氧应答元件结合进而开启下游相关基因的转录^[4]^，从而导致肺癌细胞的放化疗敏感性降低、细胞凋亡抑制、细胞周期改变、新生血管能力、侵袭转移能力增强等生物学特性变化。 

## 2 低氧对化疗的直接作用

一些化疗药物对肿瘤细胞的杀灭作用是具有氧依赖性的，它们大多通过细胞内氧化作用生成的自由基和活性氧杀伤细胞。有研究表明：抗生素类药物博来霉素在低氧状态下化疗有效率降低，可能与自由基的减少有关。Triche等^[5]^的一项研究表明，铂类化疗药物在细胞内生成自由基，自由基夺获电子后传递给氧，从而杀伤细胞。Wozniak等^[6]^在对依托泊苷的研究中发现，由于低氧状态下自由基清除剂脱氢酶抑制剂和脱氢酶底物水平提高，依托泊苷对DNA链的破坏作用较常氧状态下明显减弱。

## 3 低氧对化疗的间接作用

低氧除因氧自由基、活性氧减少，直接影响化疗药物作用机制之外，其通过诱导相关靶基因表达上调或下调，均能从不同途径导致化疗耐药。激活多药耐药基因，抑制*p53*基因表达，影响DNA损伤与修复等均是今年来研究较多的机制。

### 3.1 低氧激活多药耐药基因的作用

多药耐药（multi-drug resistance, MDR）是肿瘤细胞能够逃逸化疗药物杀伤的重要机制，也是临床肿瘤化疗失败的一个主要原因之一。研究表明低氧能够通过激活多药耐药基因的表达从而帮助细胞逃逸药物的杀伤作用。Xia等^[7]^在对肺腺癌A549细胞系的研究中发现，低氧状态下，细胞中HIF-1、P-糖蛋白和多药耐药相关蛋白的表达水平均上调，且三者具有相关性，进一步研究显示在MDR1启动子上存在功能性的HIF-1结合位点，因此HIF-1能够调控MDR1的转录活性。Gottesman等^[8]^发现低氧状态下，细胞中HIF-1、MDR1、P-糖蛋白均呈高表达状态且有相关性，证明HIF-1通过上调MDR与P-糖蛋白从而将长春碱、紫杉醇等药物泵出细胞外，使细胞内药物浓度降低，杀伤细胞能力减弱。

### 3.2 低氧抑制促凋亡基因*p53*的表达

促凋亡基因*p53*与肿瘤细胞的增殖凋亡密切相关，研究显示HIF-1α能够通过影响*p53*基因的表达，来达到抵抗诱导凋亡药物的作用。低氧通过其筛选作用诱导基因突变，如*p53*基因突变率在低氧时增加，导致细胞凋亡障碍且对诱导细胞凋亡类化疗药耐药。Hao等^[9]^研究证实，HIF-1的表达与*p53*的突变率正相关，低氧时*p53*突变的增加可以导致顺铂类药物的化疗抵抗，而HIF-1失活后，顺铂的化疗耐药性得以逆转。Sendoel等^[10]^发现，HIF-1α不仅能诱导*p53*基因突变而且还能通过抑制*p53*基因的活性来抵抗细胞凋亡，HIF-1α还可以通过增强酪氨酸酶相关蛋白2（tyrosinase-related protein）的转录活性的途径来抑制细胞凋亡。目前HIF-1α与p53相互作用的研究尚有一些未解决的问题，争议也较多。

### 3.3 低氧调节DNA的损伤与修复的作用

DNA损伤是某些药物如铂类杀伤癌细胞的作用机制，而HIF-1α可以通过调节DNA损伤和修复酶的表达水平及酶的活性，从而减弱诱导DNA损伤类化疗药物如烷化剂和铂类的化疗疗效^[11]^。Liu等^[12]^在对54例原发肺癌和正常支气管粘膜上皮的比较中发现，着色性干皮病互补组A（Xeroderma pigmentosum complementation group A, XPA）高表达，且与HIF1α呈正相关，siRNA干扰HIF-1α能明显降低XPA水平，而XPA通过促进其它DNA修复蛋白的合成的方式影响细胞对铂类药物化疗的敏感性。Lee等^[13]^在一项对以铂类为基础的小细胞肺癌一线化疗的回顾性研究中发现，ERCC1与HIF-1α表达相关，与铂类为基础的化疗呈负相关，HIF-1α低表达可能是广泛期小细胞肺癌好的预后因素。提示XPA、ERCC1均有可能成为低氧诱导铂类耐药的预测因子。依托泊苷作为小细胞肺癌的一线用药，低氧对其耐药的影响可能与DNA修复相关酶有关。Wirthne等^[14]^进行有关依托泊苷的研究时发现，HIF-1α缺失能明显增加DNA双链断裂的现象，并且相关修复DNA双链断裂损伤的蛋白激酶复合体：DNA-PKcs、Ku80和Ku70在HIF-1α在细胞中的水平明显较HIF缺失前降低。Sullivan等^[15]^的研究发现，低氧状态下，HIF-1α诱导DNA拓扑异构酶Ⅱ转录水平上调，从而增强肿瘤细胞的DNA损伤修复能力，这也可能是低氧诱导依托泊苷耐药的原因之一。

### 3.4 低氧诱导EMT的作用

上皮细胞间充质化（epithelial-mesenchymal transition, EMT）是近年来的研究热点，Matsuoka等^[16]^低氧通过TGFβ/TGFβR通路诱导EMT发生。EMT是以E-cadherin表达下调，vimentin等间充质表型标志物表达上调为标志的细胞活性转变，其在低氧诱导下不仅在肿瘤细胞的浸润、转移过程中起重要作用，在化疗耐药方面同样有重要影响。Yanu等^[17]^研究结果表明，耐奥沙利铂的结直肠癌细胞出现EMT现象，表现为细胞呈长梭形、极性丧失，瓢附性降低、伪足形成等；免疫荧光示细胞的上皮标志物E-cadherin和桥粒斑蛋白表达下调，间质标志物vimentin表达上调。研究证实PI3K通过与E-Cadherin、β-Catenin和血管内皮生长因子受体-2（vascular endothelial growth factor receptor 2, VEGFR-2）形成复合物由PI3K/AKT通路的活化参与血管内皮生长因子（vascular endothelial growth factor, VEGF）介导的内皮信号传递^[18-20]^。PI3K/AKT可能也是低氧诱导EMT的信号通路之一。苏春霞等^[21]^研究发现干性调控因子（OCT4和Nanog）及干细胞相关标志物（CD133和ABCG2）在肺癌耐药细胞株中高表达，且在肺癌组织中高于癌旁组织，进一步研究证实与E-Cadherin、N-Cadherin和化疗疗效均相关。因此干性调控因子可能也具有调控EMT作用。

### 3.5 近年新发现的其它相关通路

低氧诱导化疗耐药的机制是近年研究的热点，除以上诉述几种途径外近年来还有其它一些新发现：如低氧可以诱导产生金属硫蛋白鳌合重金属离子，从而可能导致铂类耐药^[22]^。低氧还可以通过激活其他信号通路如IGF1R/PI3K/Akt通路来抵抗凋亡从而达到抗药的效果^[23]^。Kim等^[24]^在对非小细胞肺癌A549细胞研究中发现，低氧诱导蛋白SM22α通过与IGF1Rβ相互作用活化IGF1R/PI3K/Akt通路，导致放化疗耐药。Schnitzer等^[25]^通过对肺癌A549细胞系研究发现低氧上调SphK2蛋白表达水平以及酶活性，且与S1P水平相关，同时发现用siRNA敲除SphK2可减轻细胞的化疗耐受。提高S1P数量，通过丝裂原蛋白激酶p42/44自分泌或旁分泌形式提高细胞化疗耐药能力。Brahimi-Horn等^[26]^发现线粒体中一种压力门控负离子通道（VDAC1）与代谢和凋亡有关，其缩短形式-VDAC1-ΔC具有HIF-1依赖性，其同过偶联抗凋亡己糖激酶发挥作用，从而能降低细胞对环孢素和依托泊苷的敏感性。Minakata等^[27]^在对非小细胞肺癌HCC827、PC9、HCC2935细胞系的研究中发现，PC9和HCC2935在低氧状态下对吉西他滨耐药，HIF-1α与TGFα表达上调，而HCC827引入野生型*EGFR*后在低氧下也表现出耐药。而敲除HIF-1α与*TGFα*基因则能逆转耐药。TGFα表达上调活化野生型*EGFR*可能与NSCLC吉西他滨耐药有关。

## 4 展望

低氧微环境是实体肿瘤共有的特征，虽然肿瘤低氧诱导肿瘤生物学行为恶化及化疗耐药，但同时也为肿瘤的治疗提供了一条新的思路。我们可以根据肿瘤低氧的特点指导临床化疗药物的筛选及治疗方案的优化，有利于减少治疗的盲目性，提高化疗有效率，从而提高肿瘤患者的生存期。
